# Biocompatible Polymer Nanoparticles for Drug Delivery Applications in Cancer and Neurodegenerative Disorder Therapies

**DOI:** 10.3390/jfb10010004

**Published:** 2019-01-08

**Authors:** Eleonora Calzoni, Alessio Cesaretti, Alice Polchi, Alessandro Di Michele, Brunella Tancini, Carla Emiliani

**Affiliations:** 1 Department of Chemistry, Biology and Biotechnology, Biochemistry and Molecular Biology Section, University of Perugia, Via del Giochetto, 06123 Perugia, Italy; eleonoracalzoni@gmail.com (E.C.); alicepolchi@virgilio.it (A.P.); brunella.tancini@unipg.it (B.T.); carla.emiliani@unipg.it (C.E.); 2 Centro di Eccellenza su Materiali Innovativi Nanostrutturati (CEMIN), University of Perugia, Via del Giochetto, 06123 Perugia, Italy; 3 Department of Physics and Geology, University of Perugia, via Pascoli, 06123 Perugia, Italy; alessandro.dimichele@unipg.it

**Keywords:** nanoparticles, polymers, drug delivery, cancer therapy, neurodegenerative disease therapy

## Abstract

Polymer nanoparticles (NPs) represent one of the most innovative non-invasive approaches for drug delivery applications. NPs main objective is to convey the therapeutic molecule be they drugs, proteins, or nucleic acids directly into the target organ or tissue. Many polymers are used for the synthesis of NPs and among the currently most employed materials several biocompatible synthetic polymers, namely polylactic acid (PLA), poly lactic-*co*-glycolic acid (PLGA), and polyethylene glycol (PEG), can be cited. These molecules are made of simple monomers which are naturally present in the body and therefore easily excreted without being toxic. The present review addresses the different approaches that are most commonly adopted to synthetize biocompatible NPs to date, as well as the experimental strategies designed to load them with therapeutic agents. In fact, drugs may be internalized in the NPs or physically dispersed therein. In this paper the various types of biodegradable polymer NPs will be discussed with emphasis on their applications in drug delivery. Close attention will be devoted to the treatment of cancer, where both active and passive targeting is used to enhance efficacy and reduce systemic toxicity, and to diseases affecting the central nervous system, inasmuch as NPs can be modified to target specific cells or cross membrane barriers.

## 1. Introduction

In recent years, pharmaceutical research has focused on the development of nanotechnology systems applicable in different fields of medicine, especially in the field of drug delivery. Currently, the use of biopolymers as nanoparticles (NPs) represents an alternative system with a huge potential for the targeted distribution of drugs or biological macromolecules in the body [1,2]. Biopolymer NPs can be used efficaciously to provide bioactive molecules for in vivo and in vitro applications. Nano biopolymers also find applications in the field of enzyme replacement therapy (ERT). Indeed, the possibility of using NPs constituted by biocompatible and biodegradable polymers to deliver enzymes in those tissues where they are lacking or absent represents an enormous advantage by overcoming a series of ERT problems.

More generally, nanotechnologies are an extended research field characterized by the use of materials with sizes ranging between 1 and 1000 nm [3]. The use of biopolymers in medicine has led to the definition of “polymers therapeutics” to describe different classes of nanocompounds currently in use as polymeric drugs, polymer–drug conjugates, polymer–protein conjugates, polymeric micelles, and polyplexes [4]. These systems allow active principles, peptides, and proteins as well as genes to be conveyed through localized release in the tissue of interest [5]. The use of nanometric systems allows them to easily penetrate the cells and, therefore, leads to the targeted distribution of the agent to be delivered. In the case of a therapeutic agent after administration, it is usually distributed in the body by virtue of its chemical–physical characteristics, but in a more or less systemic manner. For this reason, to reach an effective drug concentration at the site of action, administration in high and repeated doses with possible side effects is necessary. The use of biodegradable NPs is therefore aimed at “drug targeting”, that is, the selective transport of a therapeutic agent to its action site independently of the compartment or the method of administration [6]. The active principle, whatever it may be, can be dispersed, encapsulated, or adsorbed on the surface of the NPs. To date, many polymers are used for the controlled release of drugs or biological molecules. Among the most commonly used materials, polylactic acid (PLA), polyglycolic acid (PGA), and poly lactic-*co*-glycolic acid (PLGA) play a pivotal role. These polymers are extremely interesting because even if they are synthetic they are biocompatible and biodegradable [7,8,9]. In this review, the various types of biomaterials used in nanomedicine will be investigated, with a particular focus on two applications of drug delivery: (i) concerning oncological diseases that are still currently one of the main causes of death in developed countries; and, (ii) neurodegenerative diseases, by virtue of the ability of nanoparticulate systems to cross the blood–brain barrier.

## 2. Nano Biodegradable Polymers

A biodegradable polymer is a polymer that undergoes processes of degradation in vivo. Under certain specific conditions these biopolymers can arrange themselves in self-assembles of nanometric dimensions (ranging from 1 to 1000 nm) which grants them the name of nano-biopolymers [10].

**Figure 1:**
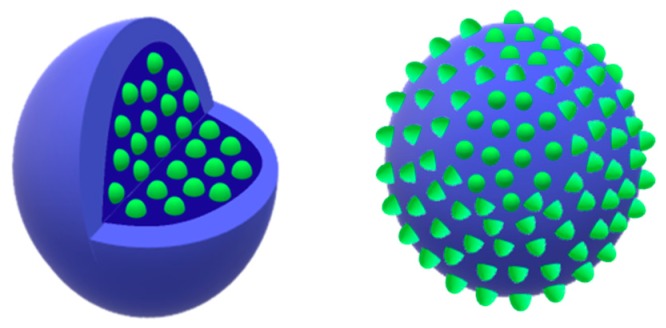
A nanocapsule and nanosphere.

Biopolymer NPs have been widely used as vehicles for drugs as they provide a series of advantages ranging from the administration of non-water-soluble drugs for the protection of unstable compounds against degradation. Indeed, NPs can be loaded with drugs either by adsorption, dispersion within the polymer matrix, or encapsulation. In this light, an obvious distinction can be drawn between nanospheres and nanocapsules (Figure 1) [10,11,12]. Nanospheres are massive colloidal particles (whose shape is not necessarily spherical) that can adsorb drug molecules on the particle surface or confine them within the particle matrix both by physical entrapment or chemical bonding. Nanocapsules can instead be seen as vesicular systems made up of a polymer shell surrounding a core cavity which typically contains either an aqueous or an oily core where the nanoparticle payload can be dissolved. An attractive alternative to nanocapsules is offered by polymersomes [10]. These aggregates are inspired by natural liposomes, and are vesicular systems made of an amphiphilic polymer bilayer (resembling the lipid bilayer of liposomes), which provides an aqueous reservoir for water-soluble drugs.

There are many techniques used to reach the desired morphology and dimensions of polymeric NPs. Broadly speaking, these methodologies can be divided into two major groups: top-down and bottom-up strategies. In the top-down approach, the NPs are generated starting from preformed polymer solutions which are later reduced in dimension down to a nanometric scale, and in the bottom-up approach monomeric polymers are led to self-assemble in order to form nanoscopic aggregates [11,13].

As for the former approach, the emulsification technique is one of the most commonly applied methodologies [11,12]. It consists of mixing two immiscible liquids, generally an aqueous solution with a non-water-miscible organic solvent, in the presence of surfactant molecules. Upon mixing, under high shear stirring, high-pressure homogenization, or ultrasonication, a simple emulsion—water-in-oil (W/O)—or a double emulsion—water-in-oil-in-water (W/O/W)—with nanoscopic dimensions can be formed. The former strategy can be applied in the case of a water-soluble polymer, which is confined in the water droplets, providing the desired NPs by cross-linking [14]. On the other hand, the double emulsion approach can be applied for the formulation of nanocapsules. For example, in a W/O/W emulsion the polymer would arrange in the oil phase surrounding the inner water pool, thus forming a nanocapsule with an aqueous core. The organic solvents employed are volatile solvents that can be easily evaporated after NPs are formed. In the past, chlorinated solvents were the natural choice, but in recent years non-chlorinated solvents such as ethyl-acetate have been preferred because of their reduced impact and toxicity [12]. After solvent evaporation, the particles are collected by ultracentrifugation and then washed to remove surfactant molecules or other additives.

However, the use of organic solvents can be disadvantageous in the case of compounds that can undergo denaturation processes. Another top-down technique, which does not require the use of any organic solvent, is extrusion. It is based on the injection of the polymer/drug mixture through a nozzle into a solution where the polymer is forced to aggregate by a change in temperature or viscosity. Depending on the nozzle size, flow rate, and solution viscosity, the properties of polymer NPs can be controlled [7,13].

On the other hand, bottom-up processes are based on the self-organization of polymer monomers as a consequence of changes in environmental parameters such as temperature, ionic strength, pH, or concentration. As a rule, they require less energy than top-down methodologies, reducing the risk of degradation, as well as allowing greater control over particle size and morphology [13]. Among bottom-up procedures, coacervation, inclusion complexation, and nanoprecipitation deserve to be mentioned.

Coacervation is a liquid–liquid phase separation process where biopolymer molecules are induced to interact, forming a separate phase (coacervate) that can encapsulate an active ingredient (typically a drug) [7,11,13]. The interactions holding together the polymer–drug complex are usually electrostatic attractions between biopolymers of opposite charges, however, hydrophobic forces and hydrogen bonding can also contribute to the formation of the coacervate. If only one type of polymer is used, this procedure is referred to as simple coacervation. When more than one type of polymer is employed a complex coacervation takes place. These soft aggregates can be cross-linked with a suitable linker (typically glutaraldehyde) in order to harden the outer shell of the particle, enhancing its stability and integrity. An appropriate drying technique is later needed to further stabilize the NPs and remove the solvent.

Inclusion complexation requires a cavity-bearing supra-molecular aggregate that can act as a host for a guest molecule. The typical interactions governing the encapsulation of the guest molecule are hydrogen bonding, van der Waals forces, and hydrophobic interactions. However, a few examples of biopolymers providing suitable cavities are available, namely β-cyclodextrins and β-lactoglobulins [11,13,15].

Nanoprecipitation is another viable technique that can be adopted in the case of hydrophobic polymers [7,10,11,12]. It uses a water-miscible solvent (i.e., acetone, ethanol, etc.) to dissolve the polymer. This polymer solution is later added to an aqueous solution (or vice versa) where the organic solvent diffuses and leads to the formation of small polymer aggregates. The organic solvent is then removed by evaporation, leaving the NPs dispersed in the aqueous phase. Surfactant can also be used in order to avoid agglomeration and coalescence of the NPs.

Another precipitation procedure goes by the name of supercritical fluid technology [11]. It resorts to a liquid or gas above its supercritical point where the polymer and drug are solubilized together. The solution is then expanded through a nozzle, leading to the fast evaporation of the solvent and the precipitation of NPs.

Irrespective of the method used for the formation of NPs, a drying technique is often required in order to remove solvent and reduce the possibility of hydrolysis for the polymer and the subsequent drug leakage. Nanocapsules and nanospheres are thus preferred in their dried form as they retain greater stability over time, even though the drying process causes additional stress for the bioactive agent. Two strategies can be followed: spray-drying or freeze-drying (also known as lyophilization) [7,11]. Spray-drying consists of the injection of the nanoparticle solution into a stream of heated air, which induces the fast solvent evaporation and the collection of dried particles. Despite being fast and economical, spray-drying of nano-emulsions and nanosuspensions allows the formation of particles with dimensions on a microscopic scale. When handling heat-sensitive materials, freeze-drying is instead chosen. Freeze-drying is a multi-step dehydration process made up of three stages: freezing, sublimation and desorption. However, high energy requirements and long drying times are the major drawbacks of this technique.

As far as the design of NPs for medical applications is concerned, one major point is the polymer biocompatibility [16]. The polymer of choice, as well as the products of its degradation, needs to be biocompatible and nontoxic to induce a minor inflammatory response. Another aspect to focus on is the biodegradability of the polymer, which must be pursued in the case of drug delivery and bone or dental implantation. Biodegradable polymers can be both natural or synthetic polymers that undergo degradation in vivo, via enzymatic or non-enzymatic routes, producing by-products further eliminated by common metabolic pathways [16,17]. As for natural biopolymers, hyaluronan, albumin, gelatin, alginate, collagen, and chitosan are some typical examples.

Among synthetic biodegradable polymers, polyethylene glycol (PEG) has been widely investigated because of its hydrophilicity, which leads to the formation of a highly water-bound barrier resulting in low cell adhesion and low protein absorption [10]. These properties are extremely appealing as they confer stealth features to PEG NPs, which can thus avoid the binding of opsonins and further clearance by the immune system. Other largely used synthetic polymers are two polyesters, polylactide acid (PLA) and polyglycolic acid (PGA), which are often used as a copolymer under the name of PLGA [7,16,18,19]. PLA and PGA differ due to the presence of a side methyl group present in PLA, which provides it with reduced crystallinity and enhanced hydrophobicity. The degradation properties of PLGA can thus be controlled by tuning the ratio between PLA and PGA units. In fact, PLGA undergoes hydrolysis of the ester linkages to form biocompatible albeit acid degradation products (lactic acid and glycolic acid) that can be easily eliminated [18]. PLGA can also be combined with hydrophilic PEG in a number of formulations to form an amphiphilic block copolymer made of regions with opposite affinity to water, augmented stealth properties and shelf stability [7].

Other synthetic biocompatible polymers that have been efficiently employed for the formation of nanocarriers are poly(ε-caprolactone) (PCL) which, at times, are conveniently used as a copolymer with lactide (PCLLA), poly-glutamic acid (PGlu), or poly(alkyl-cyanoacrylate), especially poly(butyl-cyanoacrylate) (PBCA) [20,21,22].

## 3. Biodegradable NPs in Drug Delivery Systems

The use of biodegradable NPs as molecule transporters is one of the most promising strategies for controlled-release systems (CRS). The fundamental requirement for a biomaterial to be used in this sense is its biocompatibility, that is, the ability to be metabolized without any harmful effects. Systems of this type must be able to guarantee properties such as the ability to cross the body’s anatomical barriers, typically the blood–brain barrier (BBB) or the ophthalmic barrier, controlling the concentration of the drug over time and the release of the active molecule at the site of action [23].

The release of the drug may either occur directly from NPs through diffusion, when sparingly water-soluble drugs are involved, or it may come with the dissociation of the NPs into monomeric molecules. This latter event can be triggered by local variations (particularly pH or temperature) or external stimuli (e.g., radiation or ultrasound). In so doing, the loaded NPs can fulfill its task by preventing the untimely leakage of the drug, releasing it into distinctive cellular compartments, and then producing products of degradation (the monomeric units) to be easily eliminated from the organism. The most alluring stimulus to look at is pH, as it typically varies up to three units going from blood plasma (pH = 7.4) to some cellular compartments, such as lysosomes and endosomes, where it is mildly acidic. pH-switchable NPs have been developed beginning with polymeric molecules characterized by amphoteric functionalities designed to match the pH conditions that the NPs can encounter in the human body. The drug-loaded aggregate needs to be stable during transportation in the blood plasma, i.e., at a nearly neutral pH. It also needs to turn on the drug release inside the lysosomal and endosomal compartments of a cell or within the interstitial space of solid tumors, where the pH is close to 5 [23,24,25].

These environmentally-responsive NPs have therefore earned the epithet of ‘smart’ drug delivery systems, owing to their ability to modulate and control drug release inside the targeted cells [26,27,28].

The nanoscale dimensions of NPs make them extremely promising, as they can be administered in various ways and in different regions of the body from which they will have access to target cells and tissues. Moreover, by means of functionalization processes, it is possible to evade the endosome–exosome system and to direct the drug directly to the site of action [29]. The advantages derived from the use of such systems are manifold. Firstly, it is possible to enhance the effect of the drug that will act only in the affected site and, moreover, it is possible to bypass all the drawbacks deriving from classic pharmacological treatment, for example the systemic damage caused by the inherent toxicity of the drug. Furthermore, hydrophobic molecules within this type of system are increasingly stabilized [30,31]. The drug or molecule to be conveyed, according to the synthesis technique, can be encapsulated in a nanoparticle system obtaining, in this case, nano-capsules, or dispersion in the polymer matrix to form nanospheres [32]. It is possible to create NPs of different sizes, but in general those with a diameter between 1 and 100 nm have better pharmacokinetic properties while smaller NPs are eliminated through the renal filtration system and larger NPs are absorbed by the phagocytic mononuclear system (MPS) present mainly in the liver and spleen [33].

In recent years, interest in developing NPs based on biocompatible and biodegradable polymers such as polylactic acid (PLA) polyesters and their copolymers with glycolic acid (PLGA) has increased. Despite being synthetic, these polymers are degraded in the body into oligomers and monomers that are further eliminated through the normal metabolic pathways, such as the Krebs cycle [34,35,36,37]. When polymer NPs are administered intravenously they are often subjected to the opsonization reaction, which induces their phagocytosis by the monocyte–macrophages. To overcome this problem, the particles can be coated with hydrophilic polymers such as polyethylene glycol (PEG), which prevents the recognition of the NPs by the reticuloendothelial system [38].

### 3.1. Biodegradable NPs in Cancer Therapy

Cancer is a major cause of death in developed countries. Currently, the conventional therapeutic approaches for the treatment of cancer are surgery, radiotherapy, and chemotherapy. Chemotherapy, which is currently widely used, presents high toxicity as chemotherapeutic agents inflict damage to healthy cells, thus limiting the therapeutic efficacy. Therefore, the main objective of nanomedicine in the treatment of oncological diseases is to selectively transport the drug only to cancer cells in order to improve its efficacy and reduce its toxicity. Park et al. have shown for example that doxorubicin, a potent antineoplastic drug, when encapsulated in pegylated PLGA-NPs, drastically reduces the onset of cardiomyopathies, which are one of the main side effects when using this drug systemically [39]. To date, many chemotherapeutic drugs such as doxorubicin, paclitaxel, or camptothecin have been conveyed by polymeric NPs in many types of cancer (Table 1). It should also be noted that many types of cancers are not susceptible to the classic therapeutic interventions due to alterations in the cellular mechanisms such as the base transport mediated by P-glycoprotein efflux system which is therefore responsible for the phenomenon of multidrug resistance [40].

**Figure 2:**
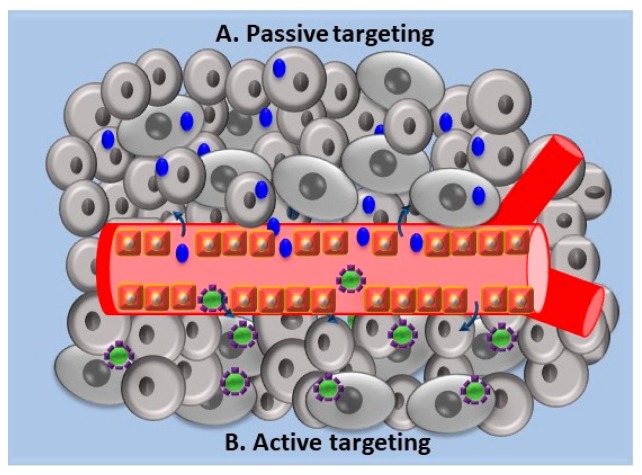
A schematic representation of passive (**A**) and active (**B**) targeting: In passive targeting NPs are released from blood vessels non-specifically to tumoral tissues by the permeation and retention effect (EPR) effect. In active targeting functionalized NPs are released into their target cells by specifically interacting with receptors found in tumoral cells.

Poly(lactide-*co*-glycolide)-d-α-tocopheryl polyethylene glycol 1000 succinate NPs were used to encapsulate doxorubicin and metformin as a P-glycoprotein inhibitor and were successfully used on breast cancer resistant cells [51]. In the treatment of neoplastic pathologies, drug delivery mediated by NPs may involve passive targeting or active targeting, with the latter remaining strictly dependent on the first [52]. Passive targeting (Figure 2A) involves the release of the drug by exploiting the characteristic vascularization of the tumor tissue that allows the passage of the molecules through convection or passive diffusion in the interstitial space and in the cells themselves. In particular, the enhanced permeation and retention effect (EPR-effect) is exploited, a process found in most cancers affecting humans. When conditions, such as an inflammatory state or hypoxia are present, the endothelial lining of blood vessels becomes more permeable, allowing the passage of molecules that accumulate in the interstitial space [53,54,55].

Active targeting (Figure 2B) is guaranteed by the adequate functionalization of the NPs through binding on the surface of specific antibodies, proteins, or peptides. In general, the ligand is chosen based on the type of receptor that is homogeneously over-expressed in tumor cells [56].

Sama et al. conducted a study in 2017 on the use of polymeric NPs able to carry epigallocatechin-3-gallate (EGCG) in prostate tumor cell models using three different types of NPs in which the active ingredient was encapsulated. They produced NPs containing non-functionalized EGCG and two different NPs appropriately tagged with small molecules able to bind to the prostate-specific membrane antigen (PSMA), which is precisely over-expressed in prostate cancer. They functionalized PLGA-PEG NPs with pseudo-mimetic dipeptide (DCL-NPs) and Asp-Glu (AG-NPs). They succeeded in demonstrating how the nanoparticle system containing EGCG has an antiproliferative effect in-vitro and was able to inhibit tumor growth on mouse models. Furthermore, for the functionalized NPs a significant increase in anti-cancer activity was observed at the site of in vivo action [57].

Aptamer-polydopamine CA(PCL-ran-PLA)-functionalized NPs have been used successfully to carry docetaxel (DTX), a potent chemotherapeutic agent in combination with a photothermal treatment in breast cancer therapy. In vitro cytotoxicity was successfully obtained by the administration of NPs and laser irradiation. In the same way, in vivo the DTX drug was found to have a better survival time and reduced side effects [58].

Another highly investigated therapeutic strategy is immunotherapy, where molecules are used to boost the immune system, making it more capable of detecting or eliminating cancer cells. In this case, immunotherapy should not be confused with vaccines that induce immunity against particular viral serogroups related to the development of tumors, as in the case of the human papillomavirus (HPV), but rather as a therapeutic strategy to be applied after the onset of the disease. The immunity in the neoplastic processes involves, firstly, the release of specific antigens by the tumor cells and the activation of the antigen-presenting cells (APCs) that, in turn, activate the effector T cells of the immune system, which can infiltrate and kill cancer cells [59]. Immunotherapy then aims at provoking specific cytotoxic T lymphocyte (CTL) responses by the activation of APCs such as dendritic cells (DCs) and macrophages. One of immunotherapy’s main objectives is to convey molecules able to activate APCs. The use of polymeric NPs for this purpose is therefore very interesting. For example, the agonists of Toll-like receptors (TLR) can stimulate dendritic cells, leading to an increase in the expression of co-stimulatory molecules and pro-inflammatory cytokines that determine the expansion of T cells. TLR 7/8 receptor agonists were encapsulated in PLGA polymeric NPs and transmitted to the lymph nodes subcutaneously, where they induced an increase in the activation of dendritic cells (DCs) and their expansion compared to the agonists administered in free form in animal models [60]. In a study conducted by Roy et al. (2010), immunotherapy with chemotherapy was combined with the use of PLGA polymeric NPs. NPs were used to carry a chemotherapeutic drug, paclitaxel (PTX), and a potent immunostimulant, SP-LPS (a non-toxic derivative of lipopolysaccharide). This strategy induced an immunostimulant and at the same time a cytotoxic effect both in vitro and in vivo, where a significant reduction in the toxicity of PTX at the systemic level has also been demonstrated [61].

The use of NPs and functionalized NPs for the treatment of cancer is, therefore, one of the main objectives of nanomedicine, as this could also be tested on molecules that are not normally used in chemotherapy because they are highly toxic but in this way may be potentially tolerable.

### 3.2. Biodegradable NPs Drug Delivery in Neurodegenerative Diseases

Neurological disorders are a large group of diseases that affect the brain and the central nervous system and include neurodegenerative, neuroinflammatory, and neoplastic diseases. The incidence of neurological disorders is increasing due to the aging of the population, and these pathologies are becoming one of the most deadly and expensive medical conditions in the world. Neurodegenerative diseases, such as Alzheimer’s (AD) and Parkinson’s (PD), are disorders characterized by an irreversible and progressive loss of neuronal cells that cause severe disability with a strong social and economic impact. At present, existing treatments can improve the symptoms but not cure these diseases due to the impairment of the functioning of various factors such as proteins and enzymes. The greatest obstacle encountered by drugs is the selectivity of the blood–brain barrier (BBB), which severely limits the number of therapeutic substances able to reach the brain to induce a positive effect. Recently, efforts have thus been made to develop systems that facilitate the passage of drugs through the BBB. Nanomedicine and in particular the targeted delivery of nanoparticle systems are gaining increasing interest among the possible strategies to be used for drug transport to the central nervous system [62,63]. The main objective of the nanoparticles is to deliver a diagnostic or therapeutic agent to a specific site. The potential benefit of site-specific delivery includes a reduction of drug dosage, an increase of the bioavailability at the desired site, and a decrease of peripheral side effects.

For this reason, in recent years, many researchers have focused their studies on the production and use of PGA, PLA, and PLGA nanoparticles that are able to cross the BBB and release drugs for the treatment of neural diseases. At first, the ability of functionalized NPs to cross the BBB was evaluated. Because of the versatility of NPs, it is possible to modify their surface during production to facilitate the interaction with different components of the BBB. Liu et al. showed that PLA-NPs that were pre-loaded with a flavonoid breviscapine were able to penetrate the BBB in a size-dependent manner, with larger particles (~300 nm) delivering more drug concentrations to the brain than smaller ones (~200 nm) [64]. In another case, trans-activating transcriptor (TAT) peptide, associated with the surface of PLA-NPs, promoted an increase in transport of the same NPs through the BBB via the bypass of efflux transporters [65]. Surface-coated PLGA-NPs with polysorbate 80 and poloaxmer 188 had shown an improved Central Nervous System (CNS) penetration [66]. Again, paclitaxel-loaded PLGA-NPs with glutathione at the surface level ameliorated the achievement of the BBB [67].

PLGA NPs have been conjugated with a glyco-heptapeptide for the transportation of loperamide and rhodamine-123 within the brain. These NPs proved to be able to efficiently pass through the BBB as their glyco-heptapeptide coating mimics the behavior of opioid peptides by absorption-mediated endocytosis [68,69].

In recent years, NP applications in the therapeutic field has expanded. In particular, researchers’ attention has shifted towards the study of functionalized NPs for the diagnosis and treatment of neurodegenerative diseases, such as AD and PD, given their high incidence in the population. In these cases, NPs are designed with a double functionalization as they are loaded with the drug to be tested and coated with specific targets for the BBB. In the scientific literature, there are plenty of papers proposing new NP formulations with different drug combinations and BBB targets. In a study carried out by Zhang and collaborators, a functionalized nanoparticle drug delivery system based on a PEGylated PLA-NPs for the treatment of AD was developed. Two targeting peptides were peculiar to these PLA-NPs, one specific for the interactions with the BBB and another showing great affinity with the Abeta 1-42 peptide. Due to the excellent Abeta 1-42 targeting effect both in vitro and in vivo, these NPs might be considerate a valuable targeting system for the diagnosis and therapy of AD [70]. In the treatment of AD and PD, PLGA-NPs are the most widely used NPs due to their properties such as controlled and sustained release, low cytotoxicity, long-standing biomedical applications, biocompatibility with tissues and cells, prolonged residence time, and targeted delivery. In work completed by Sánchez-López et al., memantine (MEM), a drug approved for the treatment of AD, was loaded in PEG-PLGA-NPs to target the BBB upon oral administration. MEM-PEG-PLGA-NPs showed a slower release profile with respect to a free drug solution because of the ability to cross the BBB both in vitro and in vivo and the reduction of beta-amyloid plaques [71]. A new drug delivery system consisting of PLGA-NPs loaded with ropinirole was developed for the treatment of PD. This system was able to revert PD-like symptoms in the animal model assayed [72]. Another approach involved the use of functionalized PLGA-NPs to reach lysosomes and restore their impaired function [73].

Modern medicine is paying a great deal of attention to the development of new drugs based on natural compounds capable of both giving early diagnosis and regressing AD. Many functionalized PLGA-NPs have therefore been loaded with curcumin, a molecule with anti-oxidant properties and with anti-amyloidogenic activity. These curcumin-PLGA-NPs have the ability to pass through the BBB and disrupt β-amyloid (Abeta) aggregates [74,75]. Recent publications suggest that PLGA-NPs functionalized with quercetin, a natural flavonoid compound, or with rosmarinic acid, a polyphenol-type carboxylic acid, may be potential candidates for AD treatment [76,77,78].

Among the diseases afflicting the CNS lysosomal storage disorders (LSDs) are worth mentioning. They consist of a group of approximately 50 pathologies caused by inherited gene mutations which occur within genes which would normally codify lysosomal enzymes. As a result, particular substrates are amassed within the lysosomes. Most LSD outbreaks occur during pediatric age with some minor exceptions that may present in adulthood. However, 75% of LSDSs involve severe neurological implications leading to physical deterioration, functional impairment, progressive neurodegeneration, and potential death [79]. As of today, there is a lack of therapies that can allow an ultimate resolution of LSDs but only symptomatic palliative and supportive treatments. During the past decades, experimental approaches aimed at restoring or substituting the deficient enzymatic activity have been formulated and tested: Enzyme replacement therapy (ERT), gene therapy, and hematopoietic stem cell transplantation are just a few remarkable examples [80,81,82,83,84,85,86,87]. Among these, ERT is the most tested approach as it is based on the periodic injection of human lysosomal enzymes produced by recombinant DNA techniques and then purified. In fact, ERT is currently used in the treatment of LSDs such as Gaucher’s disease, Fabry’s disease, mucopolysaccharidoses (MPS (type I, II and VI)), and Pompe’s disease [9,79,80,87]. Although ERT proved effective in the systemic treatment of the above-mentioned disorders, it is not ultimately decisive for most LSDs because of the inability of recombinant enzymes to cross the BBB and reach the CNS. In order to overcome this obstacle and attain therapeutic enzyme levels within the brain, several innovative strategies are now under evaluation and drug delivery mediated by NPs is certainly one of the most sought-after approaches [88,89,90]. For example, a model drug (FITC-albumin) characterized by a high molecular weight, similar to that of the enzymes lacking in MPS I and MPS II diseases, has recently been bound to PLGA NPs modified with a 7-aminoacid glycopeptide (g7), which has already proved able to deliver low molecular weight molecules across the BBB in mice. Similarly, FITC-albumin-g7-PLGA NPs were found to cross the BBB in all injected mice pointing to an efficient delivery of high molecular weight molecules into the brain by means of the investigated NP system [91]. Studies are currently underway with the aim to deliver recombinant enzymes across the BBB. As is the case with recombinant arylsulfatase A, the enzyme needed for the cure of metachromatic leukodystrophy has been efficiently attached to PLA and PLGA NPs both via high-affinity binding by the streptavidin-biotin system and via covalent bonding. However, NP-mediated brain delivery resulted in very low concentrations supposedly because of the interferences of arylsulfatase A glycoprotein with the transcytosis of the NPs across the BBB [92]. These issues imply that a great deal of effort still needs to be invested in the discovery of suitable systems able to guarantee efficient ERT. In conclusion, the use of NPs shows considerable potential for drug delivery to the CNS not only for traditional small molecule drugs, but also macromolecules such as enzymes, nucleic acids [93], proteins [94], and diagnostic agents [95]. Indeed, NPs are generally more stable and safer with respect to other nanocarrier systems such as quantum dots [21].

## 4. Conclusions and Outlooks

In this review the latest developments in the use of polymer nanocarriers as drug vehicles has been examined showing how they represent the key point for modern drug and gene delivery strategies. Various types of polymer NPs have been developed with the aim being to minimize the loss and the untimely degradation of therapeutic agents, enhance drug bioavailability, and reduce unwanted side effects by increasing drug accumulation in specific organs and tissues. Peculiar NPs can be designed that exhibit well-defined properties such as slow degradation within the human body and thus allow for the lengthening of the residence time in the blood stream, ability to cross physiological barriers (particularly the BBB), responsiveness to different stimuli (i.e., pH, temperature, etc.). From the studies reported in this review, the enormous potential of functionalized NPs stands out, as they grant the specific release of the drug by exploiting the binding between particular groups coated onto the NP surface and the cellular receptors of the target tissue or organ.

Moreover, understanding the pathophysiology of the disease under consideration is essential, inasmuch as it may allow the identification of specific cellular receptors for a highly efficient targeting outcome, which results in a focused localization of the active agent at the site of action where it is needed most and, therefore, have less detrimental effects on healthy tissues [96]. This point is especially critical in the case of CNS disorders, whose mechanisms at a molecular level are not deeply understood. Uncovering the pathobiology of a disease would make it possible to engineer functionalized NPs for highly specific targeting.

The potential impact of polymer NPs in medicine is therefore extremely promising, however, as of today some issues which prevent their widespread clinical use still need to be addressed. Although biodegradable polymers ensure the easy excretion of their oligomers through common metabolic pathways, one major problem is related to the toxicity of all components within the NP system. In particular, functionalization introduces chemical compounds the inherent toxicity of which must be reckoned with, and the use of detergents in the synthesis process must be considered [21,22,97]. Their possible toxic effects need to be deeply understood and detailed studies must be conducted. In fact, knowing the toxicity of these nanomaterials on both human health and environment is crucial, to the point where nanotoxicology is now taking hold as a brand new branch of toxicology [98]. In order for polymer NPs to come into clinical use, safety studies need to be performed for long periods of time so that the potential toxicity of nonendogenous substances that accumulate in the body can be ruled out. As a consequence, huge costs must be covered while undertaking trials aimed at verifying the applicability of the system in the human body. Another aspect to be considered is the difficulty that might be encountered when passing from laboratory to large-scale production. The scaling up of the preparation process represents a critical step that must be overcome.

In this light, polymer NPs provide fertile ground for the treatment of cancer and CNS diseases, even though long clinical trials are needed in order to put them into practical use. However, NPs provide a wide range of possibilities and the specific functionalization of the particle surface can limit their delivery into healthy cells, as well as improving their biodistribution in target organs or tissues. Engineering polymer NPs with definite surface receptors is therefore the turning point that can lead in the near future to an effective use of these versatile systems in large-scale therapeutic applications.

## Figures and Tables

**Table 1:** The combinations of anticancer drug–polymer nanoparticles (NPs) and their loading mode.

Drug	Polymer	Loading Mode	Reference
CPT	PCL-PEG	Entrapment	[41]
DOX-CUR	mPEG-PLGA-PGlu	Encapsulation	[42]
DOX-Chlorin e6-MnO_2_	PCLLA-PEG-PCLLA	Encapsulation	[43]
DOX	PLGA	Entrapment	[44]
DOX	PLGA-Cyanine5.5	Encapsulation	[45]]
DOX-Metformin	PLGA-TPGS	Encapsulation	[46]
DOX	PBCA	Encapsulation	[47]
PTX	PCL-PEG-PCL	Encapsulation	[48]
PTX	PEI-PLA	Entrapment	[49]
PTX	PLGA-PEG	Encapsulation	[50]
